# Protocol for comprehensive genotyping of nuclease, base, and prime editing outcomes using CleanFinder

**DOI:** 10.1016/j.xpro.2026.104932

**Published:** 2026-11-02

**Authors:** Jochen Dobner, Haribaskar Ramachandran, Andrea Rossi

**Affiliations:** 1 Environmental Adaptation and Cellular Resilience Laboratory, IUF-Leibniz Research Institute for Environmental Medicine, Düsseldorf, Germany; 2 Genome Engineering and Model Development Laboratory, IUF-Leibniz Research Institute for Environmental Medicine, Düsseldorf, Germany

**Keywords:** Bioinformatics, Genomics, CRISPR

## Abstract

Genome editing experiments often produce heterogeneous allele mixtures that require structured genotyping. Here, we present a protocol for analyzing amplicon sequencing data from nuclease, base, prime, and mitochondrial base editing experiments using CleanFinder. We describe steps for preparing reference sequences and FASTQ files and running browser-native or command-line analyses. We then detail interpretation of genotype distributions, repair signatures, editing efficiencies, and allelic dropout.

For complete details on the use and execution of this protocol, please refer to Ramachandran et al.^1^ and cleanfinder.org.

## Highlights

• Steps for preparing reference sequences and demultiplexed FASTQ files for analysis
• Instructions for running browser-native or command-line CleanFinder analyses
• Guidance on interpreting genotypes, repair signatures, and editing efficiencies
• Procedures for allelic dropout detection and plate-scale screening in Turbo Mode

## Abstract (editor highlights)

Publisher’s note: Undertaking any experimental protocol requires adherence to local institutional guidelines for laboratory safety and ethics.

## Graphical abstract

**Figure:**
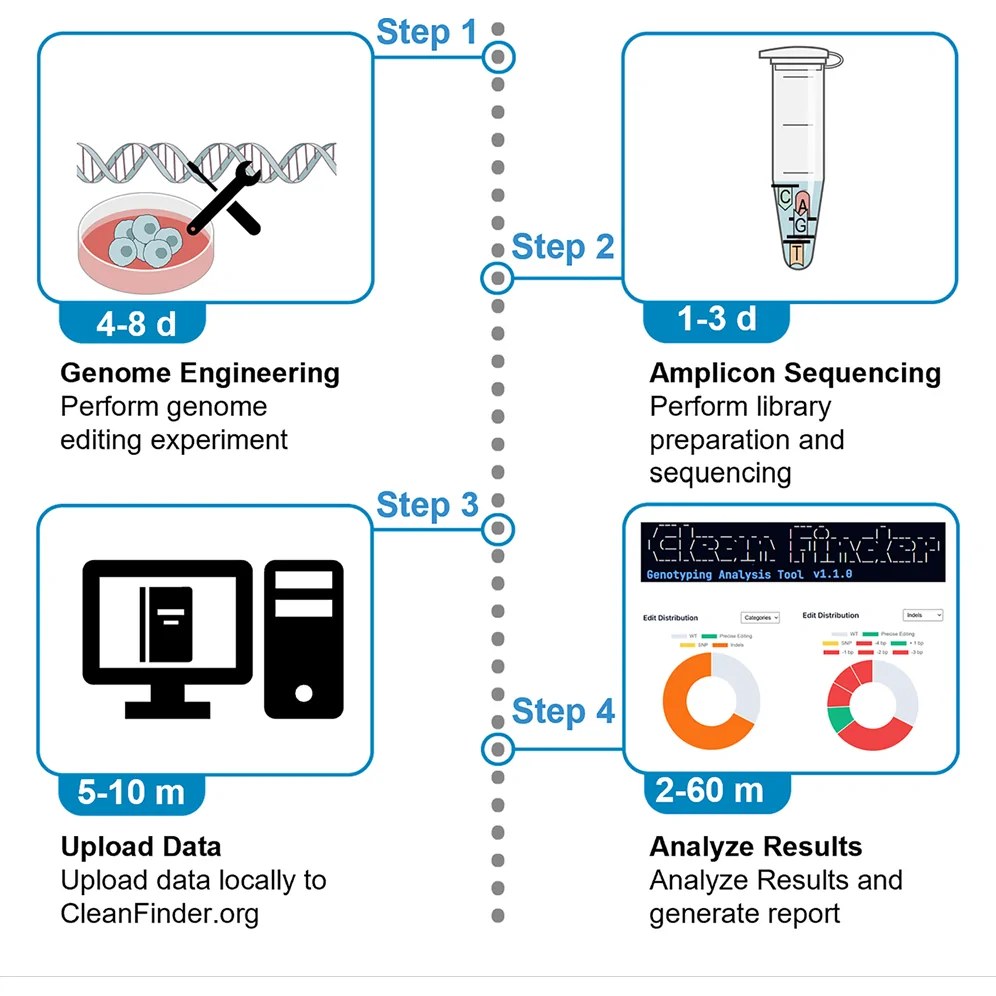

## Before you begin

Genome editing (GE) has become an indispensable approach for engineering cultured cells, including patient-derived human induced pluripotent stem cells (hiPSCs). Applications range from disease modeling to the correction of pathogenic variants. In particular, CRISPR-based technologies have enabled efficient and versatile genome manipulation, supporting the generation of gene knockouts (KOs), targeted knock-ins (KIs), and precise sequence modifications through approaches such as base editing (BE) and prime editing (PE). In addition to CRISPR nucleases, including Cas9 and Cas12/Cpf1, other programmable editing platforms such as Transcription Activator-Like Effector Nucleases (TALENs), Zinc-Finger Nucleases (ZFNs), and mitochondrial BE approaches are used to generate targeted genetic modifications. Accurate characterization of editing outcomes is essential for validating GE experiments, but genotyping edited cell populations can be challenging, particularly when samples contain heterogeneous editing outcomes, large insertions or deletions, mixed alleles, or low-frequency variants. Existing analysis workflows often require specialized bioinformatic expertise and the integration of multiple software tools, creating a bottleneck for many laboratories.

To address these challenges, we recently developed CleanFinder, a browser-native and scalable framework for the comprehensive analysis of genome editing outcomes across multiple sequencing platforms and editing modalities.^1^

This protocol assumes that a user is in principle capable of designing and executing a GE experiment, and performing next generation sequencing (NGS) of amplicons. As an example, we describe a detailed step by step protocol for performing GE and NGS of amplicons using hiPSCs. Users who already have demultiplexed FASTQ files and the corresponding reference sequences, and wish to proceed directly with data analysis, may skip the wet-lab example and begin with the CleanFinder setup and analysis sections. The same workflow can be applied to other organisms or cell types when a target amplicon sequence, expected editing design, and FASTQ files are available. CleanFinder supports Illumina, PacBio, and Oxford Nanopore Technologies (ONT) amplicon sequencing data. The protocol consists of four distinct stages: (1) generation of genome-edited cell populations, (2) amplicon library preparation and sequencing, (3) data upload and processing in CleanFinder, and (4) analysis and interpretation of genome editing outcomes.

### Innovation

CleanFinder combines browser-native genome editing analysis with an anchor-guided constrained semi-global alignment strategy. The protocol differs from conventional command-line (CLI)-only or single-sample web workflows by supporting multiple edit classes, including nuclease-induced indels (from Cas9, Cas12/Cpf1, TALENs, or zinc-finger nucleases), base substitutions, complex PE products, and (mitochondrial) BE. The workflow includes optional k-mer-guided filtering for speed, dual-reference scoring for PE, RIMA/REPAIRome annotation for deletion-associated repair signatures, and an allelic dropout module that uses heterozygous single nucleotide variations/polymorphisms (SNVs/SNPs) as endogenous allele markers. The browser implementation runs locally on the user device, reducing barriers for non-specialist users and avoiding transfer of sensitive genomic data to external servers. The Python command-line implementation enables batch processing of large datasets.

### Institutional permissions

Users must obtain all institutional and national approvals required for the biological samples used before beginning this protocol.

### Prepare genome-editing experiment information

**Timing: 15–30 min per experiment**

1. Define the type of GE experiment before starting the analysis. Record the cell type, organism, target locus, nuclease or editor system, and intended editing outcome.
2. Record the guide design used for the experiment.
  a. For nuclease-based knockout (KO) or knock-in (KI) experiments (e.g., Cas9, Cas12/Cpf1, TALENs, or ZFNs), record the guide or targeting sequence, the expected cut site, and the protospacer-adjacent motif (PAM) position where applicable.
  b. For BE experiments, record the editor type, target base, expected editing window, and expected bystander positions.
  c. For PE experiments, record the pegRNA sequence, primer binding site, reverse-transcription-template sequence, nicking guide if used, and all intended substitutions, insertions, or deletions.
3. Prepare the wild-type reference amplicon sequence in the orientation that will be used for analysis.

***Note:*** For supported organisms (human, mouse, rat, and zebrafish), reference sequences can be retrieved automatically from Ensembl using the organism, gene symbol, and guide RNA sequence.

***Note:*** In automatically retrieved genomic sequences, exonic regions are displayed in uppercase letters and intronic regions in lowercase letters to facilitate interpretation of editing outcomes and gene structure.

4. For KI or PE experiments, prepare the intended edited reference sequence.
  a. Include all expected substitutions, insertions, or deletions.
  b. Position the intended edit centrally within the edited reference sequence and retain sufficient wild-type flanking sequence on both sides of the edit (recommended: at least 5–8 nucleotides upstream and downstream).
  c. Confirm that the intended edited reference preserves the same flanking sequence as the wild-type reference.
  d. Confirm that the edited reference sequence preserves the same flanking genomic context as the wild-type reference sequence, except for the intended edit(s).
5. Define the expected analysis window around the editing site.
  a. Select 5′ and 3′ flanking regions that are outside the edit region.
  b. Avoid placing anchors within low-complexity or homopolymeric regions when possible.
  c. Record whether the analysis will use manual reference entry or reference retrieval by organism, gene symbol, and gRNA sequence.

**CRITICAL:** Use one consistent reference orientation throughout the protocol. Inconsistent reference orientation can reduce anchor detection and complicate interpretation of editing outcomes.

### Prepare amplicon sequencing files

**Timing: 15–60 min per sequencing run**

6. Generate or collect targeted amplicon sequencing data for each sample.
  a. Whenever possible, use sample-level demultiplexed FASTQ files.
  b. Maintain consistent sample identifiers, treatment groups, barcode names, and replicate annotations throughout sequencing and downstream analysis.
7. Convert raw sequencing output into FASTQ format using the workflow appropriate for the selected sequencing platform.
  ***Note:*** For most sequencing platforms, FASTQ generation and sample demultiplexing is performed automatically during or immediately after the sequencing run.
  a. If FASTQ files are not generated automatically, perform basecalling and/or demultiplexing using the manufacturer’s software or an equivalent analysis workflow of choice before proceeding with CleanFinder analysis.
8. For ONT-derived sequencing data, perform basecalling of raw POD5 files and generate FASTQ files prior to analysis.
  a. Use a high- or super-accuracy basecalling model (for example, in Dorado) when accurate genotype reconstruction is required.
  b. Lower-stringency basecalling settings may be suitable for exploratory analyses but can increase sequencing errors, particularly within repetitive and homopolymeric regions.
9. Confirm that each FASTQ file contains sufficient read depth for the expected allele frequency.
  a. Use approximately 300 reads as a practical minimum for about 95% probability of detecting a 1% allele.
  b. Use approximately 3,000 reads as a practical minimum for about 95% probability of detecting a 0.1% allele.
  c. Analyses aimed at detecting rare editing outcomes may require substantially greater sequencing depth. Additional guidance on required sequencing depth calculations for long-read amplicon sequencing can be found in.^2^
  d. For analyses involving allelic dropout, allele-specific editing, or haplotype-resolved genotyping, long-read sequencing data are recommended. Identify a stable anchor sequence within the amplicon that can be used for allele assignment. We recommend an anchor length of approximately 20 bp.
  e. If parental or unedited control samples are available, identify informative heterozygous variants within the amplicon that can be used for allele-specific assignment and allelic dropout analysis.

**CRITICAL:** ONT sequencing may exhibit reduced accuracy within homopolymeric and other repetitive regions, potentially leading to overestimation of insertion and deletion events. Editing outcomes located within or adjacent to such regions should therefore be interpreted with caution and, where possible, validated using orthogonal approaches. Super accuracy basecalling is recommended to mitigate such cases.

### Access CleanFinder and select the analysis strategy

**Timing: 5–10 min**

10. Open the browser-native CleanFinder application at cleanfinder.org in a modern web browser.
  a. Use the browser application for interactive data upload, analysis, visualization, genotype interpretation, and report generation.
  b. No local installation is required for the browser-native workflow.
  c. Use a local computer with sufficient memory for the number and size of FASTQ files analyzed.

***Note:*** CleanFinder runs locally in the user’s browser. Sequencing data are not uploaded to an external analysis server during browser-native analysis.

11. Download the CleanFinder repository if using the Python CLI or local standalone modules.
  a. Obtain the CleanFinder script and associated files from the project repository.
  b. Confirm that Python 3.7 or newer is installed before running the Python CLI.
  c. Install matplotlib if PDF report generation is required.
  d. Place the CleanFinder script, reference sequence, and FASTQ files in an accessible working directory.
12. Access the optional CleanFinder modules when required.
  a. Use Turbo Mode for rapid exploratory analysis, high-throughput screening, or plate-scale quality control.
  b. Use the Allelic Dropout module for allele-specific analysis of long-read amplicon sequencing data.
  c. Use the Genome Viewer module for locus visualization, guide RNA positioning, and predicted mutation-impact assessment.
13. Prepare the information required for analysis:
  a. A reference sequence or the information required for automatic reference retrieval (organism, gene symbol, and guide RNA sequence).
  b. Sequencing data in FASTQ format.
  c. The genome editing modality used in the experiment (e.g., nuclease editing, BE, or PE).
  d. An intended edited reference sequence for knock-in, base-editing, or prime-editing experiments, where applicable.
  e. Optional anchor, padding, and quality-filtering parameters:
    i. Anchor mismatches: the maximum number of mismatches tolerated when matching the flanking anchors. We generally leave this at zero (exact match); if anchor detection fails, for example with higher-error ONT reads, allowing one or a few mismatches can recover otherwise unaligned reads.
    ii. Padding: the length of sequence up- and downstream of the gRNA that is excluded from the analysis window. Set it according to the read or amplicon length and the size of the region to be analyzed.
    iii. Quality filtering: set the minimum per-read PHRED score. Platform-specific recommendations are provided in the following step.
14. Review sequencing quality prior to analysis.
  a. Illumina and PacBio HiFi datasets typically achieve mean PHRED quality scores of Q30–Q40 or higher and generally require minimal quality filtering (e.g., use PHRED > 35).
  b. ONT datasets typically exhibit lower PHRED quality scores than short-read or HiFi sequencing data; quality thresholds should therefore be selected according to the sequencing chemistry, basecalling model, and experimental requirements (e.g., use PHRED > 15).
  c. For applications involving low-frequency variant detection, allele-specific analysis, or precise genotype reconstruction, more stringent quality thresholds may improve analytical accuracy.
15. Define the primary objective of the analysis prior to data processing. Examples include determination of editing efficiency, genotype reconstruction, knock-in frequency, base-conversion frequency, PE outcomes, repair-pathway profiling, allele-specific editing, or allelic dropout detection.

**CRITICAL:** Ensure that the selected analysis settings are compatible with the sequencing platform and editing modality used. Excessively stringent quality filtering may reduce usable read depth, whereas overly permissive filtering can increase sequencing-error-derived artifacts, particularly in ONT-derived long-read datasets.

The preparatory workflow is summarized in Figure 1

**Figure 1:**
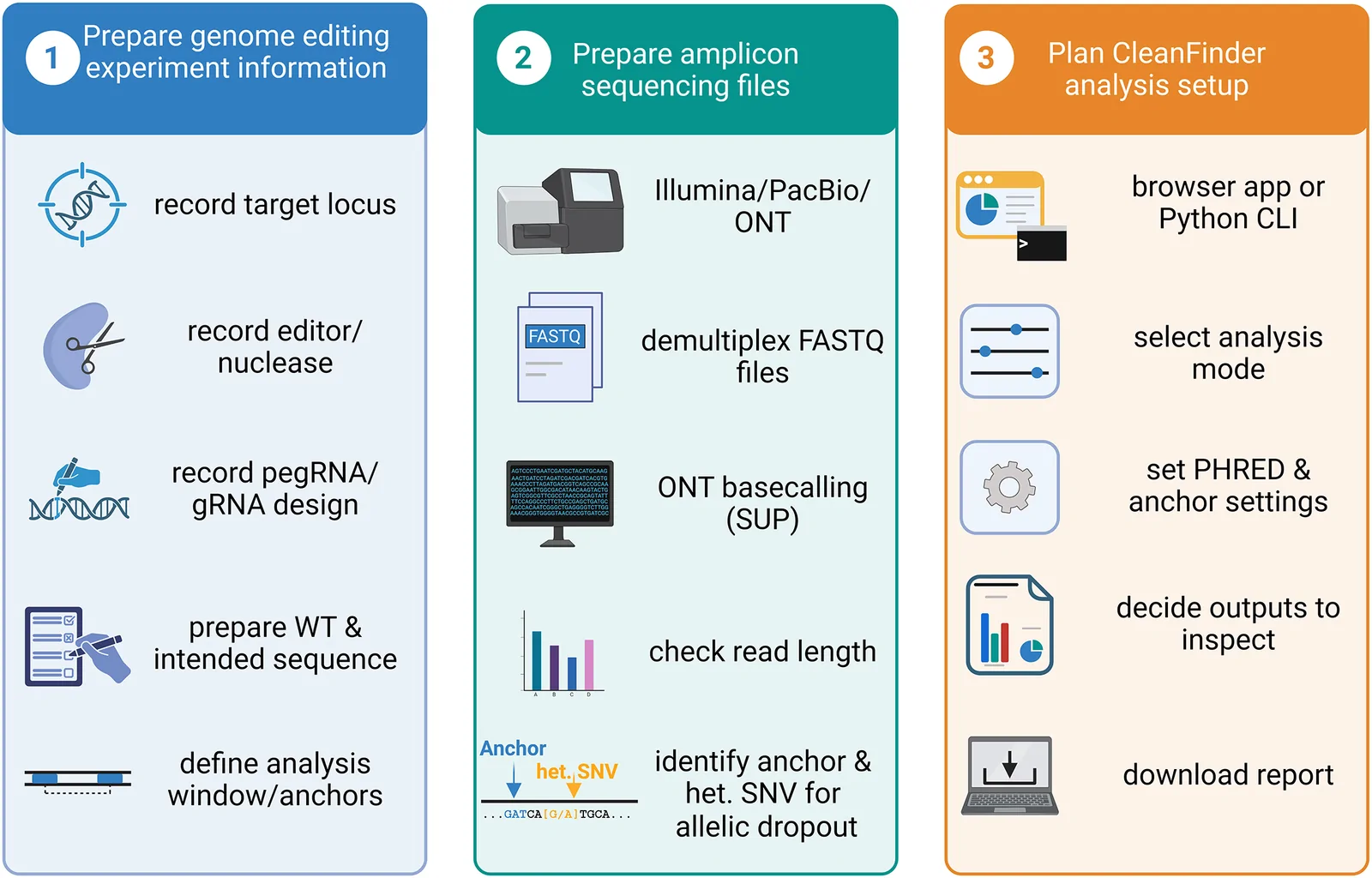
Preparation overview for CleanFinder-based genome editing analysis Schematic overview of the preparatory information and files required before starting the CleanFinder workflow. Users record the genome editing design, prepare wild-type and intended edited reference sequences, collect demultiplexed sequencing files, define anchors or analysis windows, and select the appropriate CleanFinder analysis environment. CLI: command-line interface; ONT: Oxford Nanopore Technologies; SNV: Single nucleotide variant; SUP: Super accuracy basecalling model; WT: wild-type.

### Cell culture and maintenance

**Timing: 3–5 days**

The following steps describe the preparatory steps of a representative experimental workflow used to generate input material for CleanFinder analysis. These steps are included to support reproducibility of the associated study but are not required for users who already have suitable amplicon sequencing FASTQ files.

The complete genome engineering workflow (steps 1–17) takes 4–8 days; generation of clonal lines takes a further 3–4 weeks.

***Note:*** Users who already have demultiplexed FASTQ files can skip the wet-lab steps (steps 1–29) and proceed directly to CleanFinder upload and analysis (step 30). Users starting from edited cell material can proceed to amplicon sequencing (step 18).

16. In this example, KOLF2.1J hiPSCs were maintained under feeder-free conditions using a defined pluripotent stem cell culture system.

**CRITICAL:** The quality of the starting cell population has a major impact on GE efficiency, clone recovery, and downstream genotyping results. Prior to the start of the experiment, cells should be free of contamination and exhibit the expected morphology, growth characteristics, and genomic integrity. For hiPSC-based experiments, we strongly recommend using quality-controlled cultures that have been assessed for karyotypic abnormalities, copy-number variations (CNVs), mycoplasma contamination, and pluripotency status. Experiments should be performed using healthy cultures with minimal spontaneous differentiation.

17. Prepare complete growth medium according to the manufacturer’s recommendations or established protocols for the selected cell type. For hiPSCs, prepare complete growth medium, e.g., by adding 100 mL of 5x supplement to 400 ml of mTeSR Plus medium. To mitigate risk of contamination, e.g., during enrichment of transfected cells, we recommend to add antibiotics (penicillin/streptomycin) throughout the experiment.
18. Prepare culture vessels using an appropriate extracellular matrix or coating substrate compatible with the cell type of interest. For hiPSCs, prepare extracellular matrix (e.g., Geltrex) by diluting 1:100 in ice cold DMEM-F12 medium and add 1 mL to each well of a 6-well plate and incubate for 30 min in an incubator at 37^°^ C and 5 % CO_2_.
19. Thaw cryopreserved cells and recover them according to established procedures for the selected cell type. Thaw hiPSCs at 37^°^ C, transfer the cell suspension to a 15 mL falcon tube containing 3 mL of growth medium supplemented with CEPT Cocktail (ThermoFisher), and centrifuge at 300 x g for 5 min.
20. Expand the cells under standard culture conditions until sufficient cell numbers are obtained for genome editing experiments. 24 h after plating, replace medium with fresh growth medium without CEPT.

***Note:*** We recommend to recover hiPSCs for at least two passages following thawing before initiating GE experiments.

21. Maintain and passage cells according to established protocols for the selected cell type. For clump cell passaging, aspirate the medium and wash the cells with 2 mL PBS without calcium and magnesium.
22. Monitor cell morphology, viability, and growth characteristics throughout culture. For hiPSCs, add 1 mL ReLeSR, aspirate within 1 min, and incubate cells for 5–7 min at 37° C. Monitor the cells for detachment (avoid over-incubation to prevent single cells), add 1 mL growth medium, tap the plate gently for 30–45 sec, and collect clumps into a 15 mL conical tube by carefully pipetting using a 1000 μl pipette tip.
23. Prior to genome editing, confirm that the cells meet the quality criteria required for the intended experiment.
  a. Assess cell health and the absence of contamination. For iPS cells, maintain the cells as undifferentiated colonies. Observe any signs of differentiation around the edges of a colony.
  b. Verify that cultures exhibit the expected morphology and growth characteristics. Passage the cells when they reach 60–80 % confluency.
  c. Perform cell-type-specific quality-control assays where appropriate. In the case of hiPSCs, this may include assessment of pluripotency, differentiation status, genomic stability, and mycoplasma status. In this study, hiPSC differentiation status was routinely assessed using hiPSCore.^3^

## Key resources table

**Table:** 

REAGENT or RESOURCE	SOURCE	IDENTIFIER
**Chemicals, peptides, and recombinant proteins**		

Geltrex™ Reduced-Growth Factor Basement-Membrane Matrix, LDEV-free, stem-cell qualified	Thermo Fisher Scientific	A1413301
mTeSR Plus	STEMCELL Technologies	100-0276
CEPT Cocktail Kit	R&D Systems	7991
Accutase	PAN-Biotech	P10-21500
DPBS without Ca and Mg	PAN-Biotech	P04-36500
DMEM/F12 (1:1)	PAN-Biotech	P04-41250
ReLeSR™	STEMCELL Technologies	100-0483
StemMACS CryoBrew	Miltenyi Biotec	130-109-558
Lipofectamine™ Stem Transfection Reagent	Thermo Fisher Scientific	STEM00015
Alt-R™ S.p. Cas9 Nuclease V3	Integrated DNA Technologies	1081058
Alt-R™ HDR Enhancer V2	Integrated DNA Technologies	Fisher Scientific Cat# NC2206328
Nuclease-free duplex buffer	Integrated DNA Technologies	11-01-03-01
Opti-MEM™ I Reduced Serum Medium	Thermo Fisher Scientific	31985062
Penicillin-streptomycin (10,000 U/mL, 100×)	Thermo Fisher Scientific	15140122
Fetal calf serum (FCS; fetal bovine serum, qualified)	Thermo Fisher Scientific	10270106
Proteinase K solution (20 mg/mL)	Thermo Fisher Scientific	25530049
Tween 20	Thermo Fisher Scientific	BP337-100

**Critical commercial assays**		

Blitz DNA polymerase	Klever lab	E-BLP-1B
NEBNext Library Quant Kit for Illumina	New England Biolabs	E7630S
GeneJET Gel Extraction Kit	Thermo Fisher Scientific	K0691
Native Barcoding Kit 96 V14	Oxford Nanopore Technologies	SQK-NBD114.96
384-well PCR plate	BIOplastics	B70515
Neon™ NxT 10 μL Kit	Thermo Fisher Scientific	N1096
Cell strainer, 70 μm pore size	SARSTEDT	83.3945.070

**Deposited data**		

Amplicon sequencing test data	This paper	Mendeley Data: https://doi.org/10.17632/7njkjh73pk.1

**Experimental models: Cell lines**		

Human: KOLF2.1J iPSC	The Jackson Laboratory	JIPSC001000

**Oligonucleotides**		

Cas9 guide RNAs, pegRNAs, PCR primers, and HDR donor oligonucleotides	Integrated DNA Technologies	See Tables S3 and S4 of the original study

**Recombinant DNA**		

pU6-tevopreq1-GG-acceptor	Addgene	RRID: Addgene_174038
pCMV-PEmax-P2A-GFP	Addgene	RRID:Addgene_202000
pSpCas9(BB)-2A-GFP (PX458)	Addgene	RRID:Addgene_48138

**Software and algorithms**		

CleanFinder algorithm and Python CLI	This paper	https://github.com/andrearossi-lab/CleanFinder
CleanFinder browser application	This paper	https://cleanfinder.org
Dorado	Oxford Nanopore Technologies	https://github.com/nanoporetech/dorado
Python (v3.7 or newer)	Python Software Foundation	https://www.python.org
matplotlib	Matplotlib development team	https://matplotlib.org
CHOPCHOP	Labun et al.^4^	https://doi.org/10.1093/nar/gkz365
CRISPOR	Concordet and Haeussler	https://doi.org/10.1093/nar/gky354
PE-Designer	Hwang et al.^5^	https://doi.org/10.1093/nar/gkab319
BrowserGenome	Schmid-Burgk and Hornung	https://doi.org/10.1038/nmeth.3615
RIMA v2	Taheri-Ghahfarokhi et al.^6^	https://github.com/Ghahfarokhi/RIMA
hiPSCore	Dobner et al.^3^	https://doi.org/10.1038/s41467-024-52922-1

**Other**		

Illumina MiSeq i100 Plus sequencer	Illumina	20115695
MinION Mk1B/C and Flongle flow cells	Oxford Nanopore Technologies	MinION Mk1B/C; FLO-FLG114
Qubit 4 Fluorometer	Thermo Fisher Scientific	Q33226
Neon™ NxT Electroporation System Starter Pack	Thermo Fisher Scientific	NEON1SK

## Materials and equipment

**Table:** Complete growth medium (hiPSCs)

Reagent	Final concentration	Amount
mTeSR Plus basal medium	N/A	400 mL
mTeSR Plus 5× supplement	1×	100 mL
Penicillin/streptomycin (100×)	1× (1%)	5 mL
Total	N/A	505 mL

**Table:** FACS buffer

Reagent	Final concentration	Amount
PBS (without Ca^2+^ and Mg^2+^)	N/A	49 mL
Fetal calf serum (FCS)	2% (v/v)	1 mL
CEPT cocktail	1×	As per supplier’s instructions
Total	N/A	≈50 mL

**Table:** DNA lysis buffer

Reagent	Final concentration	Amount
PBS	N/A	9.8 mL
Tween 20	1% (v/v)	100 μL
Proteinase K (20 mg/mL)	0.2 mg/mL	100 μL
Total	N/A	10 mL

**Table:** RNP mix

Reagent	Final concentration	Amount
sgRNA (50 μM)	10 μM	1 μL
Alt-R™ S.p. Cas9 Nuclease V3 (61 μM)	≈19.5 μM	1.6 μL
Opti-MEM	N/A	Up to 5 μL
Total	N/A	5 μL

**Table:** First PCR mix (targeted amplification, per reaction)

Reagent	Final concentration	Amount
Blitz PCR buffer (2.5×)	1×	2.8 μL
dNTPs (10 mM each)	0.2 mM	0.14 μL
Forward primer (2.5 μM)	0.36 μM	1 μL
Reverse primer (2.5 μM)	0.36 μM	1 μL
Blitz DNA polymerase (2 U/μL)	0.04 U/μL (0.28 U per reaction)	0.14 μL
Genomic DNA (cell lysate)	N/A	2 μL (20 ng)
Nuclease-free water	N/A	Up to 7 μL
Total	N/A	7 μL

**Table:** Index PCR mix (barcoding, per reaction)

Reagent	Final concentration	Amount
Blitz PCR buffer (2.5×)	1×	2.8 μL
dNTPs (10 mM each)	0.2 mM	0.14 μL
Forward primer (2.5 μM)	0.36 μM	1 μL
Reverse primer (2.5 μM)	0.36 μM	1 μL
Blitz DNA polymerase (2 U/μL)	0.04 U/μL (0.28 U per reaction)	0.14 μL
First PCR product	N/A	2 μL
Nuclease-free water	N/A	Up to 7 μL
Total	N/A	7 μL

***Note:*** Prepare all buffers with sterile, nuclease-free reagents and sterile-filter (0.2 μm) the FACS buffer before use. Prepare the DNA lysis buffer fresh on the day of use and add Proteinase K immediately before lysis. The first and index PCR reactions use the same mix composition and differ only in the template added.

## Step-by-step method details

The following steps describe the representative experimental workflow used to generate input material for CleanFinder analysis. These steps are included to support reproducibility of the associated study but are not required for users who already have suitable amplicon sequencing FASTQ files. The representative genome engineering workflow is illustrated in Figure 2.

***Note:*** Users who already have demultiplexed FASTQ files can skip the wet-lab steps (steps 1–29) and proceed directly to CleanFinder upload and analysis (step 30). Users starting from edited cell material can proceed to amplicon sequencing (step 18).

**Figure 2:**
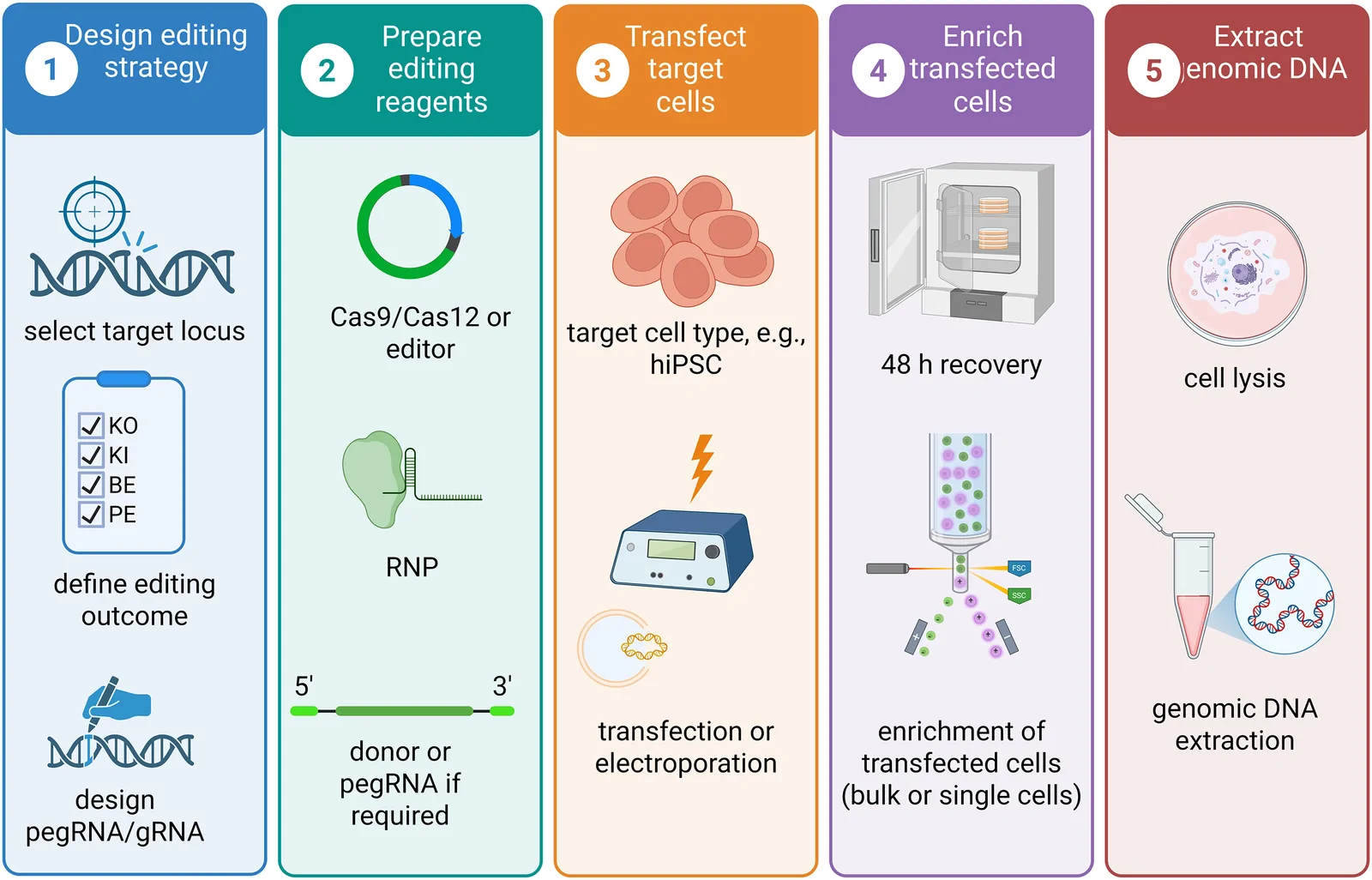
Genome engineering workflow for generating edited cell samples Schematic representation of the experimental steps preceding CleanFinder analysis. Users design the editing strategy, prepare nuclease, editor, guide RNA, pegRNA, or donor reagents, deliver the reagents to the selected cell type, recover or enrich edited cells, and extract genomic DNA for downstream amplicon sequencing.

### Design and cloning of targeting reagents

**Timing: 3–5 days**

1. Design guide RNAs (gRNAs) targeting the genomic region of interest using established prediction tools, such as CHOPCHOP,^4^ CRISPOR,^7^ or equivalent platforms.
  a. Select gRNAs based on predicted on-target activity and minimal off-target potential.
  b. For PE experiments, design PE guide RNAs (pegRNAs) using established computational predictions tools such as PE-Designer.^5^

***Note:*** We recommend selecting pegRNAs with a primer binding site (PBS) length of approximately 12–14 bp and a reverse transcription template (RTT) length of around 16–18 bp to support efficient and precise editing.

2. Introduce the selected gRNA(s) into target cells using an appropriate genome editing delivery strategy. In this protocol, gRNAs were cloned into the pSpCas9(BB)-2A-GFP (PX458) expression vector as described by Ran et al.,^8^ and plasmid constructs were verified by Sanger sequencing prior to use. Alternatively, preassembled Cas9–gRNA ribonucleoprotein (RNP) complexes may be delivered.

***Note:*** When plasmid-based delivery methods are used, clonal cell lines should be screened for potential vector integration events. We recommend performing PCR-based analysis for plasmid backbone sequences following clone isolation to confirm the absence of random genomic integration.

3. For KI experiments, design a single-stranded donor oligonucleotide (ssODN) containing the desired sequence modification and flanked by homology arms. For efficient homology-directed repair (HDR), we recommend homology arms of approximately 30–40 nucleotides on both sides of the intended edit.
  a. Whenever possible, introduce a silent mutation within the PAM, preferably at a critical PAM nucleotide, to prevent re-cleavage of the edited allele following successful genome editing.
  b. If modification of the PAM sequence is not feasible, introduce one or more silent mutations within the gRNA recognition sequence to reduce Cas nuclease binding and cleavage of the edited allele.
  c. Order the donor oligonucleotide from a commercial supplier. In this study, Alt-R™ HDR donor oligonucleotides (Integrated DNA Technologies, IDT) were used.

***Note:*** Donor oligonucleotides should be designed to avoid introducing unintended amino acid changes, cryptic splice sites, or regulatory sequence alterations whenever possible. Oligos can be designed using the CleanFinder module named Oligo (OligoDonor Designer).

4. For PE experiments, clone pegRNAs into pU6-tevopreq1-GG-acceptor as described by Doman et al.^9^

### Delivery of genome-editing reagents

**Timing: 1–3 days**

5. Prepare the cells of interest for genome editing according to established protocols for the selected cell type. Ensure that cells are healthy, actively proliferating, and at an appropriate density for the chosen delivery method.
6. Deliver the genome editing reagents using an appropriate strategy for the selected cell type and editing modality.
  a. For nuclease-mediated editing, deliver the nuclease and guide RNA as plasmid DNA, mRNA, ribonucleoprotein (RNP) complexes, viral vectors, or equivalent formats. Select the appropriate reagents for your cell type.
  b. For KI experiments, co-deliver a suitable donor template (e.g., ssODN, dsDNA donor, plasmid donor, or viral donor template) when required.
  c. Where appropriate, e.g., for KI experiments, use reagents that enhance homology-directed repair (HDR) according to the manufacturer’s recommendations (e.g., for hiPSCs Alt-R™ HDR enhancer (1:1000)).
    ***Note:*** For transfection, medium should be free of antibiotics. Chose the delivery method, e.g., lipofection, electroporation, nucleofection, or viral vectors, depending on your target cell type. ssODNs may be toxic to cells. Avoid using more than 1 μg per well of a 6-well plate.
    **Option A**: Plasmid-based delivery for knockout, knock-in and prime editing (example workflow used in this study).
  d. Plate the number of cells suitable for your target cell type. For hiPSCs, plate at-least 3 × 10^5^ cells per well of a 6-well plate in complete growth medium containing CEPT.
  e. After 24 h, aspirate the medium to remove dead cells and debris. Replenish with fresh growth medium containing CEPT.
  f. Introduce the nuclease and guide RNA expression constructs into cells using an appropriate transfection reagent or electroporation system.
    i. For KI experiments, co-deliver the donor template together with the editing construct, e.g., combine 2 μg PX458 plasmid containing sgRNA and 1 μg of Alt-RTM HDR donor oligo.
    ii. For PE, pipet 0.5 μg of pegRNA plasmid pU6-tevopreq1-GG-acceptor and 1.5 μg of pCMV-PEmax-P2A-GFP in Opti-MEM.
  g. In another reaction tube, pipette the appropriate volume of lipofectamine stem transfection reagent in of Opti-MEM. We recommend using lipofectamine reagent to plasmid ratio (3:1) or according to the manufacturer’s guidelines.
  h. Combine the contents of both tubes and incubate the mixture for 15 min at 15–25 °C. After incubation, add the transfection mix to the cells dropwise while gently swirling the plate to ensure even distribution.
  i. After 24 h, replace the medium with growth medium. Observe the cells under a microscope for cell death and fluorescence. Proceed to FACS, MACS or antibiotic selection depending upon the selection marker. We routinely use FACS (described below).
    **Option B**: Ribonucleoprotein (RNP)-based delivery.
  j. Assemble guide RNAs with recombinant nuclease protein according to the manufacturer’s recommendations.
    i. In this example, we resuspend Alt-R™ crRNA and tracrRNA in IDT duplex buffer to a final concentration of 100 μM.
    ii. In a clean PCR tube, combine crRNA and tracrRNA to generate a sgRNA at a final concentration of 50 μM.
    iii. Heat the mixture to 95 °C for 5 min and cool down to 15–25 °C at the bench.
    iv. Prepare the RNP mix as described in the materials and equipment section and incubate for 20 min at 15–25 °C.
      ***Note:*** Cas9 is supplied in glycerol-containing buffer, and may be viscous.
  k. For KI experiments, add 1 μg of the Alt-R™ HDR donor oligo to the preassembled RNP and mix gently.
    ***Note:*** ssODNs may be toxic to cells. Avoid using more than 1 μg per well of a 6-well plate.
  l. Deliver the RNP complex using a suitable electroporation or nucleofection platform optimized for the selected cell type. Here, we used the Neon™ NxT electroporation system with 10 μl tips.
    i. Generate a single-cell suspension of the KOLF2.1J cells: wash once with PBS, add 500 μl Accutase, incubate at 37 °C for 10 min, harvest the cells using PBS, centrifuge at 300 × *g* for 5 min, and resuspend the cells in electroporation buffer.
    ii. Transfer RNP-cell mixture to the tip, and electroporate at 1,300 V, 10 ms, and a single pulse.
  m. Allow cells to recover under standard culture conditions before proceeding with downstream selection or genotyping. For hiPSCs, we immediately resuspend cells in growth medium containing CEPT, and transfer them to Geltrex-coated wells of a 6-well plate.
7. Enrich or select edited cells if desired.
  a. Fluorescent reporters may be used for enrichment by FACS (e.g., fluorescent Cas9 or tracrRNA).
  b. Magnetic enrichment (MACS) may be used when compatible markers are available.^10^
  c. Antibiotic selection may be applied when resistance markers are incorporated into the editing construct.
  d. Alternative enrichment strategies may be used according to the experimental design.
8. Verify successful reagent delivery using an appropriate readout, such as fluorescence reporter expression, antibiotic resistance, molecular genotyping, or equivalent methods.

**CRITICAL:** GE efficiency depends strongly on cell health, reagent quality, and delivery conditions. Human iPSCs should actively proliferate, form compact, multi-layered colonies with defined edges and a high nucleus to cytoplasm ratio, show no signs of contamination and less than 5 % spontaneous differentiation. For delivery, antibiotic-free medium should be used during transfection. Preferably, RNPs should be used over plasmids for increased efficiency of editing hiPSCs. Optimization of the delivery method for the cell type of interest is often required before large-scale editing experiments are initiated.

### Fluorescence-activated cell sorting

**Timing: 2–3 h**

This section describes how to enrich transfected cells by fluorescence-activated cell sorting and, if required, recover bulk-edited populations or isolate single-cell-derived clones for downstream genotyping.

**CRITICAL:** Always include antibiotics during cell sorting to prevent contamination.

9. Prepare the cells for FACS 24 h post transfection. A reduced percentage of GFP positive cells are typically observed, if FACS is performed at later timepoints, i.e., after 48–72hrs.
10. Prepare fresh FACS buffer (PBS without magnesium and calcium supplemented with 2 % FCS and CEPT; sterile-filtered (0.2 μM, Sarstedt)).
11. Obtain single cell solutions as described above.
12. Mix the cell suspension thoroughly and pass through a 70 μm cell strainer to remove cell aggregates and obtain a single cell suspension.
13. Centrifuge at 300 × *g* for 5 min and resuspend the cell pellet with 300 μl of FACS buffer.
14. Sort the GFP-positive cells and collect the cells in growth medium containing CEPT.
15. Centrifuge at 300 × *g* for 5 min and resuspend the cell pellet in 2 mL of growth medium containing CEPT and plate the cells into a well of a 6-well plate.
16. After 24 h exchange medium to growth medium, and allow the FACS-sorted bulk cells grow until they are 60–80 % confluent.
17. Proceed to bulk analysis or/and isolate single clones as described below:
  a. Prepare a single cell suspension as described above, and pass through 70 μm cell strainer to remove aggregates. Alternatively, single-sort cells into wells of a 96-well plate during enrichment of transfected cells (steps 12–15 above).
  b. Seed cells into Geltrex-coated wells of a 96-well plate at a calculated density of 1.5 cells per well in 50 μl of growth medium containing CEPT, and incubate at 37°C.
  c. Starting after 48 h post plating, exchange growth medium every other day with fresh growth medium until visible colony formation (usually at least 2 weeks).
  d. Once colonies reach 60% confluency, prepare lysis (for genotyping) and maintenance (for expansion) plates by equally splitting the colonies into separate 96 well plates.

### Amplicon sequencing

**Timing: 2–3 days**

This section describes how to lyse edited cells, amplify the target locus, add sample-specific barcodes and sequencing adapters, and generate FASTQ files for CleanFinder analysis. The amplicon sequencing workflow is illustrated in Figure 3.

18. Design primers for targeted amplicon sequencing that flank the genome-edited region and generate an amplicon suitable for the chosen sequencing platform. Ensure that the expected amplicon spans the editing site and is compatible with downstream library preparation and sequencing requirements.

***Note:*** Primer design can be performed using standard primer design software or online tools, such as BrowserGenome^11^ (https://www.browsergenome.org/).

19. Prepare DNA lysis buffer consisting of PBS supplemented with 1% (v/v) Tween 20 and Proteinase K (20 mg/mL stock) diluted to a final concentration of 0.2 mg/mL. Add 50 μL of lysis buffer to each well of a 96-well plate containing cell pellets and incubate at 56 °C for 1 h.
20. Following lysis, incubate the plate at 95°C for 15 min to inactivate Proteinase K.

***Note:*** Incomplete inactivation of Proteinase K may inhibit downstream PCR amplification.

21. For the first PCR step of Illumina library preparation, assemble the targeted amplification reaction using the first PCR mix given in the materials and equipment section.

***Note:*** For ONT, refer to the library preparation steps of the Native Barcoding Kit 96 V14 (SQK-NBD114.96): Ligation sequencing amplicons – Native Barcoding Kit 96 V14.

22. Perform the PCR using the following setup:

**Table:** 

Steps	Temperature	Time	Cycles
Initial denaturation	98 °C	1 min	1
Denaturation	98 °C	20 s	19 cycles
Annealing	60 °C	20 s	–
Extension	72 °C	30 s	–
Final extension	72 °C	4 min	1
Hold	12 °C	Indefinite	1

**Figure 3:**
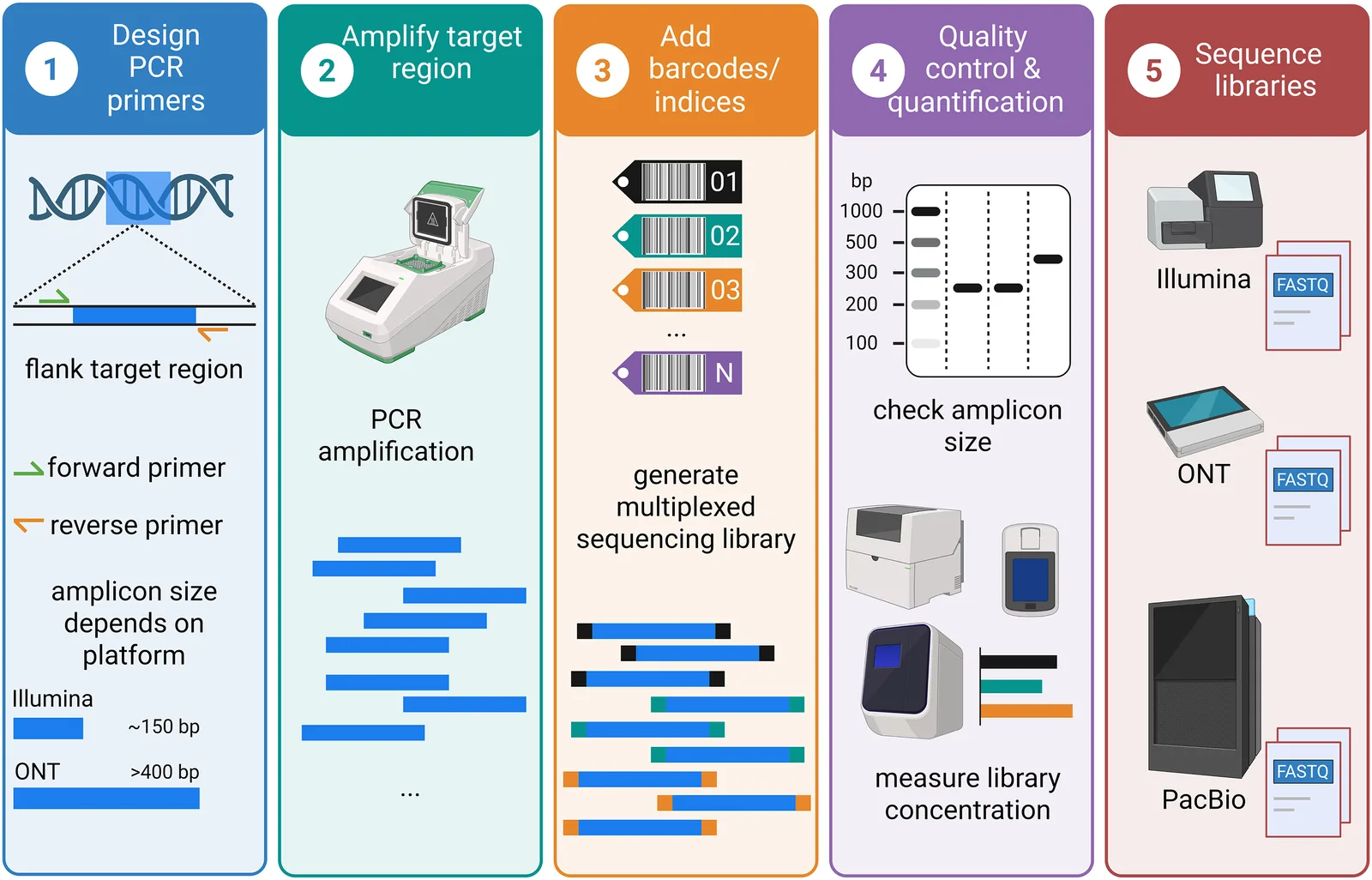
Amplicon sequencing workflow for genome editing readout Schematic overview of targeted amplicon sequencing preparation. Users design primers flanking the edited region, amplify the target locus, add sample barcodes or indexes, perform library quality control and quantification, and sequence the libraries using Illumina, ONT, PacBio, or another FASTQ-generating sequencing platform.

23. Incorporate sample-specific barcodes and sequencing adapters into the amplicons to enable multiplexed sequencing. In this example, barcodes were introduced by a second-round PCR (index PCR) using indexed primers and the index PCR mix given in the materials and equipment section. Alternatively, use a commercial library preparation kit that incorporates sample barcodes and sequencing adapters according to the manufacturer’s instructions.

***Optional:*** Check PCR products on a 1.5 % agarose gel to confirm size and specificity.

24. Perform the PCR using the same conditions used for the first PCR described before.
25. Pool the indexed PCR products and assess library quality by agarose gel electrophoresis. For high-throughput experiments, individual reactions may be pooled prior to purification.
26. Using a clean scalpel, excise the DNA fragment corresponding to the expected library size from the agarose gel. In our workflow, the final indexed amplicon library was approximately 450 bp in length.
27. Purify the DNA using a gel extraction kit according to the manufacturer’s instructions.
28. Quantify the purified library using a fluorometric assay (e.g., Qubit 4 Fluorometer) or a qPCR-based library quantification method (e.g., the NEBNext Library Quant Kit for Illumina). qPCR-based quantification is generally recommended for accurate estimation of sequenceable library molecules prior to sequencing. Prepare and load the library according to the recommendations of the selected sequencing platform and library preparation workflow.

***Note:*** Amplicon sequencing libraries are typically low-diversity libraries due to the high sequence similarity among reads. In this study, libraries were sequenced on an Illumina MiSeq i100 Plus instrument, which demonstrated robust performance with low-diversity amplicon libraries. Library loading concentrations and sequencing parameters should be optimized according to the manufacturer's current recommendations for the instrument and chemistry used (the optimal value depends on the clustering behaviour of the instrument, and we recommend loading between 60 and 80 pM of the final library as a starting point; see Illumina's loading concentration recommendations for the MiSeq i100 Series).

29. Once the run is complete, extract the FASTQ files according to the manufacturer’s instructions, and proceed with downstream analysis genome editing using CleanFinder.

### Upload data and analysis

**Timing: 5–60 min**

This section describes how to define the analysis window, provide the reference sequence, upload FASTQ files, configure the appropriate analysis mode, and start CleanFinder processing. The CleanFinder analysis and result interpretation workflow is summarized in Figure 4.

***Note:*** This step includes selecting the files for analysis and setting the parameters. Timing depends on the number and size of files uploaded, and the local computational power.

30. Specify the window of data analysis.
  a. Provide reference DNA sequence and specify anchors.
    i. Manual: paste reference sequence manually.
    ii. Ensembl: retrieve the reference sequence from the database. Select the species of the amplicon of interest, provide the gene symbol, enter the gRNA sequence used to position the analysis anchors, and click Fetch to obtain a centered reference sequence.
      ***Note:*** In references retrieved from Ensembl, exonic sequences are shown in capital letters and intronic sequences in lowercase letters, so exon and intron boundaries can be read directly from the retrieved reference.
      ***Optional:*** KI oligo: specify knock-in sequence for targeted GE experiments
  b. 5′ and 3′ padding: specify sequence length up- and downstream of the provided gRNA which is ignored for the actual analysis window.
31. Prepare the sequencing data for upload.
  ***Note:*** CleanFinder has been validated with data in FASTQ format from long-read sequencers (Oxford Nanopore Technologies, PacBio) and short-read sequencers (Illumina), and is expected to work with amplicon sequencing data in FASTQ format from any other NGS method.
  a. Select files for upload by drag & drop or browse (“Sequencing Reads”).
32. Set parameters for sequencing data analysis (“Configuration”).
  a. Select “Analysis Mode” depending on GE experiment setup. This can be either one of:
    i. “Standard (Cas9)” (default) for CRISPR/Cas9-based and other double-strand-break nuclease KO experiments (e.g., TALENs, ZFNs).
    ii. “Standard (Cas12/Cpf1)” for CRISPR/Cas12-based KO experiments.
    iii. “Prime Editing” for resolving complex mixtures of PE outcomes.
    iv. “Base Editing” for resolving single nucleotide substitutions introduced by BE.
      ***Note:*** Although the modes are named for the most common systems (Cas9, Cas12/Cpf1, prime editing, and base editing), CleanFinder is editor-agnostic and can resolve essentially any genome-engineering outcome. We have, for example, applied it to mitochondrial DNA (mtDNA) BE. For non-standard editors, select the closest mode and adapt the alignment scoring matrix under Advanced Alignment Parameters.
  b. Specify “Advanced Alignment Parameters” to fine-tune analysis:
    i. Set settings for the allowed number of mismatches for anchor search.
    ii. Adjust the alignment scoring matrix: match score, mismatch penalty, gap-opening penalty, and gap-extension penalty. Tuning these values tailors the alignment to the expected edit type — higher gap penalties suppress spurious indels when scoring substitutions (e.g., nuclear or mtDNA BE), whereas lower gap penalties favour the detection of larger insertions and deletions.
    iii. Activate a speed-boosting mode for faster analysis. Two platform-specific k-mer-seeded options are available, ONT Fast (K-mer Seeded) and Illumina Fast (K-mer Seeded). Each derives a short seed from the 5′ anchor (a 13-mer for ONT and a 10-mer for Illumina/standard) and rapidly skips any read that does not contain this seed on either strand, so that full alignment is performed only on candidate on-target reads. This greatly increases throughput, but reads carrying sequencing errors within the seed region (more common in noisy ONT data) may be skipped, which can lower sensitivity; leave these options off when maximal sensitivity or precision is required.
    iv. Set the pre-analysis read quality filter by specifying a minimum per-read PHRED score (default: 15). Reads whose mean per-base PHRED quality falls below this threshold are discarded before alignment; as a guideline, use approximately 25 or higher for Illumina and PacBio data and approximately 15 or lower for ONT data.
33. Start analysis by activating the button “Start Analysis”.

**Figure 4:**
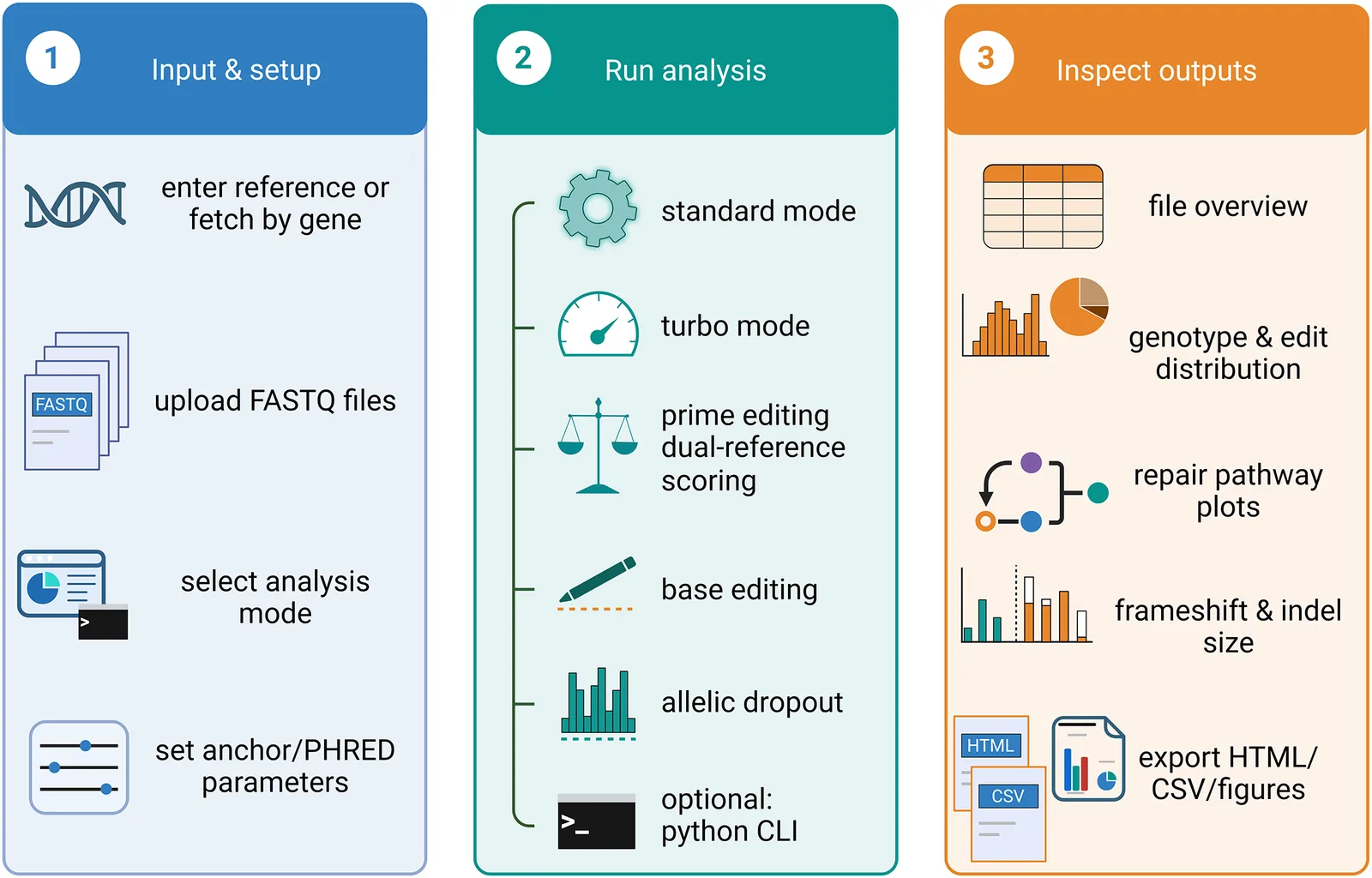
CleanFinder upload, analysis, and result interpretation workflow Schematic overview of the CleanFinder analysis workflow. Users enter or retrieve the reference sequence, upload FASTQ files, select the analysis mode, configure anchor and quality parameters, run the analysis using standard mode, Turbo mode, or the Python CLI, and inspect outputs including file overview, edit distribution, genotype distribution, repair pathway plots, Genome Viewer, and exported reports.

### Analyze results

**Timing: 5–15 min**

This section describes how to inspect CleanFinder outputs, including aligned-read metrics, edit distributions, genotype-level calls, repair-pathway annotations, clone summaries, and downloadable reports.

***Note:*** Timing depends on the user, but obtaining comprehensive results is fast. CleanFinder offers extensive comprehensive analysis and visualization of GE outcomes.

34. “File Overview” provides an overview of the GE outcomes of analyzed files in either grid or heatmap view, both aggregated as well as by sample.
35. “Edit Distribution” provides detailed distribution of identified genomic edits either grouped in detail by base changes (“indels”) or by the type of edit (“category”).
36. Information on total number of reads aligned after filtering, editing efficiency in %, percentage of out-of-frame mutations, in-frame mutations, and precise editing (PE or KI) are provided.
37. “Genotype Distribution” provides detailed per sample information on the type of GE outcomes identified, the exact aligned sequence, and RIMA-based analysis^6^ of the type of repair pathway involved in repairing a DNA double-strand break. This analysis can be filtered for the minimum number of reads for an outcome to be taken into account, the minimal relative occurrence in the sample, and the minimal PHRED score to be taken into account.

***Note:*** Adjusting these parameters does not require additional computational power.

38. Below the detailed genotype information, graphical visualizations on indel distribution, frameshift distribution, microhomology length, repair pathway, and PAM proximity of deletions, are provided.

***Note:*** For each genotype, CleanFinder displays a set of badges summarizing the edit and its likely repair outcome. The edit-type badge labels the read as wild-type (WT), substitution (SNP), insertion (INS), deletion (DEL), knock-in / homology-directed repair (HDR/KI; read matches the intended knock-in template), base edit (BE; intended base conversion without a length change), or prime edit (PE; read matches the intended prime-edit sequence). A frame badge marks indels as In-Frame when the net length change is a multiple of three and Out-of-Frame (frameshift) otherwise; wild-type and substitution-only reads carry no frame badge. For deletions, a repair-pathway badge is assigned from the microhomology detected at the deletion junction: non-homologous end joining (NHEJ) when junction microhomology is absent or shorter than 2 bp, and microhomology-mediated end joining (MMEJ; also termed MHEJ or alternative end joining) when the junction carries microhomology of at least 2 bp. The length of the microhomology tract is reported as a microhomology (MH) badge (e.g., MH-1, MH-2, MH-3, and longer), and the position of each deletion relative to the cut site and PAM is shown. This Rational Indel Meta-Analysis (RIMA v2)-based annotation,^6^ together with the REPAIRome framework^12^ (RIMA v2 available at https://github.com/Ghahfarokhi/RIMA), distinguishes precise from error-prone repair and supports interpretation of editing fidelity and reproducibility.

39. Below, a concise summary of the individual clones is provided. Downloads of a HTML report, a spreadsheet summary, and of the visualizations, can be performed from this tab.

***Note:*** In the clone summary, a clone is flagged as a KO only when it is out-of-frame and two distinct edited sequences are detected (a biallelic, out-of-frame genotype). To avoid mistaking allelic dropout for a homozygous KO in diploid cells, a single out-of-frame sequence is not called a KO. The two alleles are expected within an approximately 70:30 ratio; if one allele falls below ∼30% of the reads, the clone is not flagged as KO.

This section introduces optional CleanFinder modules that extend the standard workflow for rapid plate-scale screening, allele-specific dropout analysis, and gene or guide-RNA visualization.

### Rapid screening with Turbo Mode

**Timing: 2–15 min for a 96-well plate**

This section describes how to use Turbo Mode for rapid primary screening of plate-scale FASTQ datasets before reanalyzing selected samples with the standard browser workflow or Python CLI.

***Note:*** Turbo Mode (TurboMode.html) is a lightweight, high-throughput alternative to the standard analysis, optimized for the rapid screening of large experiments, e.g., 96- or 384-well plates. Whereas the standard browser application and the Python CLI align every read with a rigorous glocal (anchor-guided, constrained semi-global) algorithm, Turbo Mode uses a faster heuristic alignment. This increases throughput at the cost of some precision when calling complex or non-standard variants.

40. Open TurboMode.html, paste the WT reference sequence and, optionally, the gRNA sequence (used for highlighting).
41. Name the FASTQ files by well position for automatic plate assignment: the pattern is a letter A–H followed by a number 1–12 at the start of the filename (e.g., A1_VHL.fastq, B3-sample.fq, C12.fastq). Files without a recognizable well position are assigned sequentially (A1, A2, …, H12).
42. Upload one or more FASTQ files (or paste sequences directly, one per line), then click Run Analysis.
43. Inspect the plate view. Results are displayed as a 96-well grid (A1–H12) in which each well is represented by a pie chart of the allele distribution, colour-coded as wild-type (green), HDR (purple), in-frame indel (blue), out-of-frame indel (red), and substitution/SNP (amber).
44. Wells with fewer than 100 aligned reads are shown dimmed (reduced opacity, dashed border) to flag data insufficient for reliable genotyping; hover over a well to see its exact read count.
45. Click any well to view its detailed allele breakdown below the plate, including the allele table (genotype, alignment, type, size delta, and read count) and an indel-size distribution chart. Use the Min Reads filter (default 100) to hide low-frequency alleles.

**CRITICAL:** Turbo Mode is intended for rapid categorization and primary screening, not for the detailed reconstruction of complex mixtures. Reanalyze prioritized or ambiguous samples with the standard browser application or the Python CLI, which use the full glocal alignment.

### Genotyping analysis with the Python command-line interface

**Timing: 5–30 min, depending on file size and batch number**

**Table:** 

Flag	Default	Category	Description
-r, --ref	required	Input	Reference sequence: FASTA file or plain DNA string (overridden by --gene)
-q, --reads	required	Input	FASTQ file(s) with sequencing reads; supports multiple space-separated files or wildcards
-g, --grna	—	Input	gRNA sequence (without PAM) used to anchor the analysis window
--gene	—	Input	Fetch reference from Ensembl by gene symbol (requires internet; overrides -r)
--species	homo_sapiens	Input	Ensembl species for --gene (aliases: human, mouse, rat, zebrafish)
--mode	cas9	Logic	Analysis mode: cas9, cas12, prime, or base
--hdr	—	Logic	Expected HDR/knock-in template sequence
--pe-edit	—	Logic	Expected prime-edit sequence (required for prime mode)
--be-conversion	—	Logic	Base-conversion pattern, e.g., “C>T” (required for base mode)
--min-phred	0	Filter	Minimum average Phred score per read (0–40)
--max-mismatches	2	Filter	Maximum mismatches allowed in anchor regions
--anchor-len	20	Filter	Length of flanking anchors (bp)
--window-size	40	Filter	Window size on each side of the gRNA (bp; max ∼200)
-o, --output	results.csv	Output	Output CSV path, or directory for batch mode
--top	15	Output	Number of top genotypes to display in console/PDF
--show-all	—	Output	Display all detected genotypes (overrides --top)
--no-alignment	—	Output	Disable the alignment visualization in the console
--quiet	—	Output	Print only summary statistics (useful for large batches)
--pdf	—	Output	Generate a publication-quality PDF report (requires matplotlib)
--batch	—	Batch	Path to a CSV config file for processing heterogeneous samples

This section describes how to run the alignment-based CleanFinder workflow from the command line for large batches, files that exceed browser memory, headless systems, or automated bioinformatics pipelines.

The Python CLI (cleanfinder.py) runs the same alignment-based analysis engine as the browser application from a terminal. Use it for large batches, files exceeding browser memory, on headless servers, or for integration into automated bioinformatics pipelines. The browser application and the CLI use the identical glocal (constrained semi-global) alignment workflow and therefore return equivalent genotype calls.

Full command-line reference:

46. Confirm that Python 3.7 or newer is installed. Core analysis requires no external packages (Python Standard Library only); PDF report generation additionally requires matplotlib. No installation is needed beyond the single cleanfinder.py script.
47. Place the reference (a FASTA file or a plain-text sequence) and one or more FASTQ files in an accessible directory.
48. Run a single-sample analysis with the reference (-r), reads (-q), and gRNA (-g):

python cleanfinder.py -r ref.fa -q sample_A1.fastq -g TCTGCAGAATGTCAAAG

49. Select the analysis mode with --mode (cas9 is the default; cas12, prime, base). Prime editing requires --pe-edit, base editing requires --be-conversion, and knock-in detection uses --hdr:

python cleanfinder.py -r ref.fa -q reads.fq -g ATCGATCGATCG --mode cas9

python cleanfinder.py -r ref.fa -q reads.fq --mode prime --pe-edit GCTAGCTAG --window-size 60

python cleanfinder.py -r ref.fa -q reads.fq --mode base --be-conversion “C>T”

***Note:*** Quote the base-conversion pattern (for example, “C>T”) so that the shell does not interpret > as output redirection.

50. Tune quality and window behaviour with --min-phred (read quality filter), --max-mismatches (anchor tolerance), --anchor-len, and --window-size. Filtering low-quality reads is recommended for ONT data to prevent erroneous mutation calls:

python cleanfinder.py -r ref.fa -q sample.fq -g GRNA --min-phred 25 --window-size 60

51. Optionally retrieve the reference directly from Ensembl by gene symbol with --gene (and --species) instead of providing -r. CleanFinder prints “Reference: N bp” to confirm what was fetched:

python cleanfinder.py --gene VHL --species homo_sapiens -g TCTGCAGAATGTCAAAG -q reads.fastq

52. Analyze multiple files that share one reference and gRNA (e.g., a 96-well plate) by passing several FASTQ files or a wildcard ∗. A detailed report is produced for each file and a multi-well summary table comparing editing efficiency is appended at the end:

python cleanfinder.py -r ref.fa -g GRNA -q ∗.fastq -o summary.csv

53. For heterogeneous experiments where each sample has a different reference, gRNA, or mode, use batch mode with a CSV configuration file (columns: sample_name, fastq, reference, grna, mode):

python cleanfinder.py --batch config.csv -o results_folder/

54. Control the outputs: -o sets the CSV path (or output directory in batch mode); --top N or --show-all set how many alleles are displayed; --quiet and --no-alignment reduce console output; and --pdf produces a publication-quality PDF report with a summary dashboard and per-allele alignment figures:

python cleanfinder.py -r ref.fa -q reads.fastq -g GRNA -o results.csv --pdf --top 30

55. Inspect the outputs. The console report shows summary statistics (total, aligned, unaligned, WT %, edited %), genotype and frame bar charts, MMEJ-vs-NHEJ repair-pathway distribution, and an alignment view with markers (∗ = mismatch/SNP, + = insertion, ˆ = deletion). The results.csv lists every detected genotype with its type, size Δ, frame, aligned sequences, read count, and percentage. The optional PDF adds a colour-coded summary dashboard and detailed alignment blocks for the most abundant alleles.

***Note:*** The --top and --show-all flags affect only the console and PDF display. The results.csv always contains every detected genotype regardless of these flags.

### Allelic dropout analysis

**Timing: 5–20 min per sample**

This section describes how to use long-read amplicon data and heterozygous SNVs/SNPs to assess whether an apparently homozygous editing outcome reflects true biallelic editing or loss of one allele.

The Allelic Dropout analyzer detects heterozygous SNVs/SNPs in long-read amplicon data to determine whether an apparently homozygous editing outcome reflects true biallelic editing or the loss (“dropout”) of one allele, for example through a large deletion or rearrangement that would otherwise be misread as a homozygous knockout. Open the Allelic Dropout in any modern browser; no installation or server is required.

56. Open the Allelic Dropout module and upload the sequencing file (FASTQ). The module is optimized for long-read (ONT or PacBio) data; ONT super-accuracy basecalling is recommended.
57. Define a stable anchor sequence of approximately 15–20 bp. It must be unique, present in all reads, and located in a conserved region away from the expected editing site and candidate SNV/SNP (we recommend an anchor at least 100–200 bp from the expected edit and any candidate variant).
58. Provide the wild-type reference by pasting the amplicon sequence or using the Fetch from Gene Symbol tool. Supplying a gRNA during fetch centers the reference on the region of interest.
59. Set the parameters: scan window (bases searched on either side of the anchor; default 500 bp), minimum coverage (reads required to call a SNV/SNP; default 500), and the minimum/maximum allele frequency expected for a heterozygous SNV/SNP (default 30–70%).
60. Click “Run Analysis”. The tool aligns reads around the stable anchor, builds a base-frequency matrix for every position in the scan window, and identifies high-coverage positions that show two distinct bases within the target frequency range.
61. Read the call. BIALLELIC (green) indicates a high-confidence heterozygous SNV/SNP, indicating both alleles are present. MONOALLELIC (amber) indicates that no heterozygous SNV/SNP was found, which may reflect allelic dropout or a naturally homozygous sample. ERROR (red) indicates that the analysis failed, typically due to poor anchor matching or insufficient coverage.
62. Review the SNV/SNP table: the position relative to the anchor, the identity and frequency of the two observed alleles, and a visual snippet of the reference context around each SNP.

**CRITICAL:** Place the anchor outside the edited region. An anchor that overlaps the edit can fail to match dropout-affected reads and bias the call.

***Note:*** A monoallelic call alone is not sufficient to prove an allelic dropout event. Where parental or unedited control data are available, confirm against known heterozygous SNVs/SNPs in the amplicon before concluding that an allele has been lost.

### Genome Viewer

**Timing: 5–15 min**

This section describes how to visualize gene structure, locate guide RNAs, predict nuclease cut sites, and preview the coding- or protein-level consequences of candidate indels.

The Genome Viewer module is a standalone, browser-based companion tool for visualizing a gene, locating a gRNA on the transcript, predicting Cas9 and Cas12a cut sites, and simulating the protein-level impact of a CRISPR indel—all without running the command-line pipeline. It is particularly useful when planning functional gene knockouts. Open genome_viewer.html directly in your browser; it fetches gene structure and sequence data live from Ensembl and UCSC.

63. Open genome viewer module and enter the HGNC gene symbol (e.g., *VHL, BRCA1, SNAI1*) and, optionally, the protospacer (gRNA) sequence without PAM.
64. Click Draw structure to render the canonical RefSeq transcript as an exon–intron diagram. Use the zoom, Fit to View, and Labels controls; the mouse wheel zooms, and Shift+wheel or click-drag pans the diagram.
65. Optionally click View in UCSC to open the locus in an embedded UCSC Genome Browser view (hg38).
66. With a gRNA entered, the viewer overlays the protospacer span (amber), the Cas9 blunt double-strand break 3 bp upstream of the NGG PAM (red dashed line), and the Cas12a staggered cut zone 18–23 nt from the 5′-TTTV PAM-proximal end (orange shading). An info panel reports the exact genomic coordinates of the gRNA and both cut sites and whether the gRNA was found on the forward or reverse strand.

***Note:*** If the gRNA spans an exon–intron boundary it may not be found in the genomic window; in that case enter the CDS cut position manually in the mutation-impact section.

67. Click Show CDS & Protein and select a transcript (canonical is recommended) to display the full coding sequence and translated protein and to auto-detect the cut position.
68. In the Mutation Impact Preview, choose the nuclease (Cas9 or Cas12a), click Auto-detect from gRNA to fill the CDS cut position, select an indel size using the presets (−1, −2, −4, −5, +1, +3) or a custom value, and click Simulate.
69. Read the simulation result: the frame call (In-Frame or Out-of-Frame/frameshift); a nonsense-mediated decay (NMD) badge predicting whether a premature stop codon is NMD-sensitive or NMD-escaped under the 50–55 nt exon-junction-complex rule^13^; summary metrics (indel size, first affected codon, new stop codon position, and percentage of wild-type protein retained); and a side-by-side, color-coded alignment of the wild-type and mutant protein sequences.

***Note:*** The NMD prediction approximates the 50–55 nt rule using an estimated last exon-junction position. For a definitive NMD assessment, validate with experimental data or a dedicated splice-aware tool.

### Companion sequence-design tools (optional)

**Timing: 5–10 min**

CleanFinder includes two companion utilities that support experiment design but are not required for genotyping analysis. The OligoDonor Designer module aids the design of single-stranded HDR donor oligonucleotides for knock-in experiments: it loads the target transcript, locates the gRNA, lets the user define the intended protein-level edit, and proposes a donor with homology arms and silent mutations that prevent re-cleavage of the edited allele, while reporting the selected strand and melting temperature. The Reverse Complement module provides quick sequence operations, including reverse, complement, reverse-complement, and DNA/RNA conversion, together with basic primer-design helpers and a codon reference table. Both tools are convenient for preparing reagents and reference sequences but play no role in the CleanFinder analysis itself.

## Expected outcomes

CleanFinder generates interactive and exportable summaries of GE outcomes from targeted amplicon sequencing. For indel-based experiments using CRISPR-Cas9/Cas12 or other programmable nucleases (e.g., TALENs, ZFNs), users should obtain total aligned reads, editing efficiency, WT frequency, genotype distributions, insertion and deletion sizes, in-frame and out-of-frame classifications, and repair pathway annotations. For BE experiments, users should obtain position-specific nucleotide conversion frequencies and the ability to separate intended edits from bystander substitutions and residual indels. For mitochondrial BE, users should obtain heteroplasmy estimates for edited mitochondrial reads.

For PE experiments, users should obtain dual-reference classifications that separate wild-type reads, precise intended edits, bystander variants, incomplete integrations, and compound alleles. For screening-scale experiments, exported tables should allow normalization to controls and ranking of candidate modulators. For allelic dropout analysis, users should obtain a biallelic or monoallelic classification based on heterozygous SNV/SNP representation, together with read coverage and allele-frequency metrics.

The expected output files include interactive HTML reports, CSV or Excel-compatible tables, alignment summaries, genotype-frequency tables, and publication-ready graphics generated from the browser application. The Python CLI generates tabular outputs suitable for batch processing and downstream statistical analysis.

## Limitations

CleanFinder depends on correct target sequence information, adequate read depth, and suitable anchor placement. Incorrect reference pasting, primer trimming artifacts, or missing target sequence can reduce alignment rates or misclassify alleles. ONT sequencing can introduce platform-specific basecalling biases, especially in homopolymeric regions resulting in artificially elevated indel rates. Genotypes called within homopolymers should therefore be interpreted with caution, and confirmed by orthogonal analysis, when necessary, e.g., by performing sequencing using Illumina, PacBio HiFi, Sanger sequencing, or by clone-level validation.

The Turbo mode is optimized for rapid categorization and primary screening rather than detailed reconstruction of complex alleles. It may be less suitable for reads containing concurrent deletions, insertions, substitutions, long templated PE insertions, or shifted integration boundaries. The allelic dropout module requires informative heterozygous SNVs/SNPs and is most powerful when long reads phase distant SNVs/SNPs and editing events on the same molecule/read. A single-dose compound screen can nominate candidate modulators, but it does not establish potency, specificity, or mechanism without secondary validation.

## Troubleshooting

### Problem 1

Low read depth or many reads fail preprocessing (related to steps 18–29).

### Potential solution

Increase library input, when possible, repeat library quantification, review barcode demultiplexing, and resequence samples that do not meet the detection threshold required for the expected allele frequency. As a guide, approximately 300 reads are required for about 95% probability of detecting a 1% allele, while approximately 3,000 reads are required for a 0.1% allele. To correctly represent editing populations more reads are necessary. If many reads fail anchor detection, confirm that the reference sequence matches the amplicon orientation and primer design. Check whether primers, adaptors, or unexpected trimming changed the read boundaries. Move the anchor away from low-quality termini or highly edited regions and allow a bounded number of mismatches if sequencing quality is variable.

### Problem 2

Edited alleles are not detected or are inconsistently classified (related to steps 30–39).

### Potential solution

Verify the gRNA, pegRNA, editing window, and intended-edited reference. Inspect raw read quality and confirm that the sample was successfully edited by an orthogonal assay if available. For prime editing, ensure that the intended reference includes all planned nucleotide changes and that the wild-type reference has not accidentally been edited. If prime editing products are split into many similar intermediate alleles, use the standard alignment workflow rather than Turbo mode, inspect alignment strings for shifted integration boundaries or partial reverse-transcription-template integration, and collapse only biologically equivalent alleles after manual review. For indel calls in homopolymeric regions, use ONT super accuracy basecalling, compare with Illumina or PacBio HiFi data, avoid overinterpreting single-genotype calls inside long homopolymers, and flag homopolymer-overlapping deletions in the report.

### Problem 3

Analysis is slow, memory-limited, or allelic dropout results are inconclusive (related to steps 30–69).

### Potential solution

Use k-mer filtering, reduce the number of simultaneously loaded files, analyze samples in batches, or switch to the Python CLI. For exploratory multiwell screening, use Turbo mode first and reanalyze prioritized samples with standard mode. For inconclusive allelic dropout results, increase long-read depth, choose an anchor that robustly captures the full amplicon, and verify the presence of heterozygous SNPs in the parental or unedited sample. If no informative SNP is present, use an orthogonal assay such as PCR across the deletion, ddPCR, SNP array, or whole-genome sequencing.

## Resource availability

### Lead contact

Requests for further information, resources, and reagents should be directed to and will be fulfilled by the lead contact, Andrea Rossi (andrea.rossi@iuf-duesseldorf.de).

### Technical contact

Technical questions on executing this protocol should be directed to and will be answered by the technical contact, Andrea Rossi (andrea.rossi@iuf-duesseldorf.de).

### Materials availability

All unique and stable reagents generated in the associated study, including gRNAs, cell lines, and genetically modified cell lines, are available from the lead contact upon reasonable request.

### Data and code availability

The datasets analyzed in this protocol were generated in the original study, and their availability is described there; the amplicon sequencing data are deposited in Mendeley Data and are publicly available at https://doi.org/10.17632/7njkjh73pk.1. All additional data reported in the original study will be shared by the lead contact upon request. The original CleanFinder code has been deposited on GitHub and is publicly available at https://github.com/andrearossi-lab/CleanFinder. The released version of record accompanying the original publication, test data, and the Python package are available at cleanfinder.org. The version of CleanFinder described in this protocol is Genotyping Analysis Tool v.1.1.0; the current release is v.1.3.0. A changelog documenting all improvements over earlier releases, including substantially faster analysis through k-mer-guided pre-alignment filtering, is available within the software (the Info panel at cleanfinder.org and in the GitHub repository). CleanFinder is updated approximately once per month.

## Acknowledgments

This project was directly supported by the 10.13039/501100001659Deutsche Forschungsgemeinschaft (DFG) (RO5380/10-1 to A.R.) and the AFM-Téléthon (25179 to A.R.).

The Environmental Adaptation and Cellular Resilience Laboratory and the Genome Engineering and Model Development Laboratory are also funded by the DFG (RO5380/1-1, RO5380/2-1, RO5380/13-1, and RO5380/14-1 to A.R.), von Hippel-Lindau Betroffene Familien e.V. through a grant awarded to A.R., the EU and North Rhine-Westphalia start-up grant (IN-SU-3-001 to A.R. and J.D.), and the Leibniz Competition (SAW) Cooperative Excellence project (K642/2024 to A.R.).

The IUF-Leibniz Research Institute for Environmental Medicine is funded by the federal and state governments, the Ministry of Culture and Science of North Rhine-Westphalia (MKW), and the Federal Ministry of Research, Technology and Space (BMFTR).

## Author contributions

Conceptualization, A.R.; methodology and development, A.R.; investigation, H.R.; visualization, J.D.; writing – original draft, J.D., H.R., and A.R.; manuscript preparation, J.D. and A.R.; supervision, funding, and review and editing, A.R. All authors reviewed and approved the manuscript.

## Declaration of interests

The authors declare no competing interests.
